# How Breath-Control Can Change Your Life: A Systematic Review on Psycho-Physiological Correlates of Slow Breathing

**DOI:** 10.3389/fnhum.2018.00353

**Published:** 2018-09-07

**Authors:** Andrea Zaccaro, Andrea Piarulli, Marco Laurino, Erika Garbella, Danilo Menicucci, Bruno Neri, Angelo Gemignani

**Affiliations:** ^1^Department of Surgical, Medical, Molecular and Critical Area Pathology, University of Pisa, Pisa, Italy; ^2^Coma Science Group, GIGA Consciousness, University of Liège, Liège, Belgium; ^3^National Research Council, Institute of Clinical Physiology, Pisa, Italy; ^4^Nuovo Ospedale degli Infermi, Biella, Italy; ^5^Department of Information Engineering, University of Pisa, Pisa, Italy; ^6^Azienda Ospedaliero-Universitaria Pisana, Pisa, Italy

**Keywords:** slow breathing, breath-control, pranayama, paced breathing, EEG, fMRI, HRV, psychophysiology

## Abstract

**Background:** The psycho-physiological changes in brain-body interaction observed in most of meditative and relaxing practices rely on voluntary slowing down of breath frequency. However, the identification of mechanisms linking breath control to its psychophysiological effects is still under debate. This systematic review is aimed at unveiling psychophysiological mechanisms underlying slow breathing techniques (<10 breaths/minute) and their effects on healthy subjects.

**Methods:** A systematic search of MEDLINE and SCOPUS databases, using keywords related to both breathing techniques and to their psychophysiological outcomes, focusing on cardio-respiratory and central nervous system, has been conducted. From a pool of 2,461 abstracts only 15 articles met eligibility criteria and were included in the review. The present systematic review follows the Preferred Reporting Items for Systematic Reviews and Meta-Analyses (PRISMA) guidelines.

**Results:** The main effects of slow breathing techniques cover autonomic and central nervous systems activities as well as the psychological status. Slow breathing techniques promote autonomic changes increasing Heart Rate Variability and Respiratory Sinus Arrhythmia paralleled by Central Nervous System (CNS) activity modifications. EEG studies show an increase in alpha and a decrease in theta power. Anatomically, the only available fMRI study highlights increased activity in cortical (e.g., prefrontal, motor, and parietal cortices) and subcortical (e.g., pons, thalamus, sub-parabrachial nucleus, periaqueductal gray, and hypothalamus) structures. Psychological/behavioral outputs related to the abovementioned changes are increased comfort, relaxation, pleasantness, vigor and alertness, and reduced symptoms of arousal, anxiety, depression, anger, and confusion.

**Conclusions:** Slow breathing techniques act enhancing autonomic, cerebral and psychological flexibility in a scenario of mutual interactions: we found evidence of links between parasympathetic activity (increased HRV and LF power), CNS activities (increased EEG alpha power and decreased EEG theta power) related to emotional control and psychological well-being in healthy subjects. Our hypothesis considers two different mechanisms for explaining psychophysiological changes induced by voluntary control of slow breathing: one is related to a voluntary regulation of internal bodily states (enteroception), the other is associated to the role of mechanoceptors within the nasal vault in translating slow breathing in a modulation of olfactory bulb activity, which in turn tunes the activity of the entire cortical mantle.

## Introduction

### Rationale

Breathing is intimately linked with mental functions. In the millenary eastern tradition, the act of breathing is an essential aspect of most meditative practices, and it is considered a crucial factor for reaching the meditative state of consciousness, or “Samadhi” (Patanjali, Yoga Sutras). The breath is called “Prana,” which means both “breath” and “energy” (i.e., the conscious field that permeates the whole universe). “Prana-Yama” (literally, “the stop/control,” but also “the rising/expansion of breath”) is a set of breathing techniques that aims at directly and consciously regulating one or more parameters of respiration (e.g., frequency, deepness, inspiration/expiration ratio). Pranayama is primarily related to yoga practice, but it is also part of several meditative practices (Jerath et al., 2006).

A growing number of scientific studies in the field of Contemplative Neuroscience (Thompson, 2009) are reporting accurate descriptions of mental and somatic effects elicited by meditation. The large number of published studies has led to the need of reviews and meta-analyses with the aim of eliminating possible confounding factors, stemming from the heterogeneity of the investigated meditative techniques, differences among experimental designs across studies, and from the overuse of subjective assessments in meditative effects' evaluation. The purpose of these scientific efforts is threefold: (i) building a shared and standardized taxonomy of meditation techniques (Lutz et al., 2007; Ospina et al., 2007; Nash and Newberg, 2013; Van Dam et al., 2018); (ii) identifying psychophysiological correlates of meditation and of meditation-related practices (Sperduti et al., 2012; Fox et al., 2014; Boccia et al., 2015; Lomas et al., 2015; Tang et al., 2015; Gotink et al., 2016); (iii) assessing the effectiveness of meditative techniques as treatments in different preclinical and clinical conditions (Ospina et al., 2007; Chiesa et al., 2011; Creswell, 2017).

Heuristically, it is commonly acknowledged that breathing techniques are profoundly intermingled with cognitive aspects of meditation, and in eastern culture, their role for achieving altered states of consciousness is undisputed. A common belief of western culture is that breathing control has beneficial effects on health status, such as wellness, relaxation and stress reduction (nearly a million results googling the keywords “pranayama,” and “wellness,” or “stress”). Nevertheless, western science has paid little attention to the investigation of the effects of pure breathing control on neural correlates of consciousness, and on specific mental functions.

Returning on meditative practices, the main issue in unveiling the basic mechanisms underlying their effects is to disentangle those related to breathing control from those associated with non-respiratory cognitive components such as focused attention and mental imagery.

To our best knowledge, only ten dedicated reviews tackle the effects of Pranayama, without succeeding in the identification of a common psychophysiological model (Srinivasan, 1991; Brown and Gerbarg, 2005a; Singh et al., 2009; Sengupta, 2012; Brown et al., 2013; Nivethitha et al., 2016; Brandani et al., 2017; Russo et al., 2017; Kuppusamy et al., 2018; Saoji et al., 2018). Some authors have even attempted at modeling the effects of Pranayama (Brown and Gerbarg, 2005b; Jerath et al., 2006; Brown et al., 2013; Gard et al., 2014; Riley and Park, 2015; Schmalzl et al., 2015), but a general consensus on the identification of the psychophysiological mediators that link Pranayama to its beneficial outcomes is still lacking. Other authors, focusing their attention on the benefits of Pranayama in different pathological conditions (e.g., asthma, hypertension, insomnia, anxiety, and depression), involuntarily added further confounding factors for the identification of Pranayama's basic mechanisms: the main issue is the lack of a consistent knowledge of physiological mechanisms leading to the beneficial effects of Pranayama and, from a clinical standpoint, their interaction with pathophysiological ones underlying the abovementioned diseases.

In western culture, breathing techniques were developed independently from any religious or spiritual belief or purpose, and nowadays are mainly used for therapeutic aims (e.g., biofeedback, progressive relaxation, autogenic training). These breathing techniques are often referred to as paced breathing (Stancák et al., 1993) and are based on slowing down the breath frequency. Paced breathing has been associated with relaxation and well-being (Jerath et al., 2015), while fast breathing has been often mutually linked to anxiety and stress (Homma and Masaoka, 2008).

To our best knowledge, both for Pranayama and paced breathing, no systematic review focusing either on their basic mechanisms or on their effects in healthy subjects has ever been published (but see Lehrer and Gevirtz, 2014; Mather and Thayer, 2018).

### Objectives and research question

The aim of this review is the identification of common psychophysiological mechanisms underlying the beneficial effects of slow breathing techniques (<10 breath per minute) by systematically reviewing the scientific literature. Only studies involving healthy humans, avoiding thus possible confounding effects due to pathological conditions, and dealing with the voluntary modulation of breathing (Pranayama and paced breathing) were included. It is in fact crucial to distinguish between slow breathing techniques, and other techniques that simply direct attention to the act of breathing (e.g., breath awareness, breath counting) or slow down breath as a consequence of other attentional practices (e.g., Transcendental Meditation, Nidra Yoga). Studies based on self-reports instruments alone were not included, as their reliability is severely weakened by the absence of objective measures, a major and common problem when dealing with contemplative sciences (Schmalzl et al., 2015). We focused on studies investigating both changes of physiological parameters related to central and/or autonomic nervous systems activity in slow breathing techniques trials, and their relationships with behavioral outputs.

The physiological parameters taken into account in this systematic review are brain activity, investigated by Electroencephalography (EEG) and functional Magnetic Resonance Imaging (fMRI), and autonomic activity, studied by Heart Rate Variability (HRV), Respiratory Sinus Arrhythmia (RSA), and Cardio-Respiratory Synchronization.

To develop an effective search strategy, we adopted the Population, Intervention, Comparison, Outcomes and Study Design (PICOS) worksheet (see Methods and Table 1).

**Table 1:** PICOS.

**Parameter**	**Inclusion criteria**	**Exclusion criteria**
Population	Healthy humans. Expert or naïve for breathing techniques	Young (<18 years) and/or old (>65 years) subjects. The population comprised any chronic or acute pathology
Intervention	Any technique of breath control that directly slows the breath down to 10 breaths per minute.	Breathing paced at a frequency higher than 10 b/min. Techniques that do not comprise an active modulation of breathing. Mixed techniques which includes psycho-physical practices other than breath regulation (e.g., meditation, visualization, yoga postures). Protocols which includes active emotional induction (e.g., fear, anger or stress induction)
Comparison	Comparison techniques (e.g., spontaneous breathing) or control groups (active interventions, no-intervention)	
Outcomes	Physiological outcome related to cardio-respiratory system or central nervous system (i.e., EEG, fMRI, HRV, RSA, and Cardio-Respiratory Synchronization), together with a psychological/behavioral outcome (assessed with a psychometric quantitative approach) Measured during slow breathing techniques (state effect), immediately afterwards (state effect), or after a long-term intervention (trait effect)	Physiological parameter of no interest. Measured only a physiological or a psychological/behavioral parameter alone
Study design	Within subjects, cross sectional, randomized controlled, longitudinal, pre-post	Case reports. Lack of rigorous description of the experimental set-up and methodology, impeding replicability

## Methods

### Search strategy

This systematic review followed the Preferred Reporting Items for Systematic Reviews and Meta-Analyses (PRISMA) guidelines (Moher et al., 2009). PRISMA comprises a 27-item checklist that has to be completed in order to improve quality of systematic reviews (Moher et al., 2009). The check-list is reported in Supplementary Table 1. The protocol of this systematic review has been submitted for registration in PROSPERO database, international prospective register for systematic reviews, with ID number 105537 (https://www.crd.york.ac.uk/prospero/).

A systematic search of MEDLINE and SCOPUS electronic databases has been performed. The initial search was conducted in March 2016, while the final search was carried out in April 2018. Boolean operators “AND” and “OR” were applied for combining keywords related to breathing techniques and to their physiological outcomes. A search example for the slow breathing techniques is the combination of the following keywords: “Pranayama” OR “Breathing Technique” OR “Breathing Exercise” OR “Paced Breathing” OR “Controlled Breathing” OR “Slow Breathing” OR “Deep Breathing” OR “Metronome Breathing” OR “Yoga” OR “Heart Rate Variability Biofeedback.” A search example for the physiological outcomes is the combination of the following keywords: “Cardiorespiratory Synchronization” OR “Cardiorespiratory Coupling” OR “Cardiorespiratory Interaction” OR “Cardiorespiratory Coherence” OR “Respiratory Sinus Arrhythmia” OR “Heart Rate Variability” OR “Electroencephalogram” OR “Magnetic Resonance Imaging” OR “Functional Connectivity”. We searched both for extended names and their acronyms. The complete list of search keywords is reported in Appendix 1.

### Study design

Following PICOS strategy, we defined the inclusion and exclusion criteria (Table 1). Studies identified from the literature search were included if:

- They were conducted on healthy humans (both expert or naïve for breathing techniques)
- Any technique of breath control that directly slows the breath down to 10 breaths per minute was used
- A comparison technique (e.g., spontaneous breathing) or control groups (active interventions, no-intervention) was included
- A physiological outcome was measured, related to cardio-respiratory system or central nervous system (i.e., EEG, fMRI, HRV, RSA, and Cardio-Respiratory Synchronization), together with a psychological/behavioral outcome (assessed with a psychometric quantitative approach).

We considered eligible for the inclusion all studies assessing physiological parameters during slow breathing techniques (state effect), immediately after (state effect), and after long-term interventions (trait effect).

Studies identified from the literature search were excluded if:

- Young (<18 years) and/or old (>65 years) subjects were recruited
- The population comprised any chronic or acute pathology
- Breathing was paced at a frequency higher than 10 b/min
- Techniques do not comprise an active and direct modulation of breathing, investigating instead “passive” breathing techniques (i.e., breathing modulation as a by-product of other meditation/attentional/yoga techniques, e.g., Breath Awareness, Nidra Yoga, Transcendental Meditation, Tai Chi Chuan, QiGong)
- The intervention was not limited to breathing exercises, but included also other techniques as meditation, visualization, or required specific yoga postures (e.g., specific position and movements as in Hatha Yoga)
- The protocol used active emotional induction (e.g., fear, anger or stress induction)
- Measured a physiological parameter of no interest, or measured only a physiological or a psychological/behavioral parameter alone
- They were case reports
- The applied methodologies and/or techniques were not well-described or replicable
- Not published in a peer-reviewed journal
- Not available in full-text and/or in English language.

## Results

### Flow diagram

**Figure 1:**
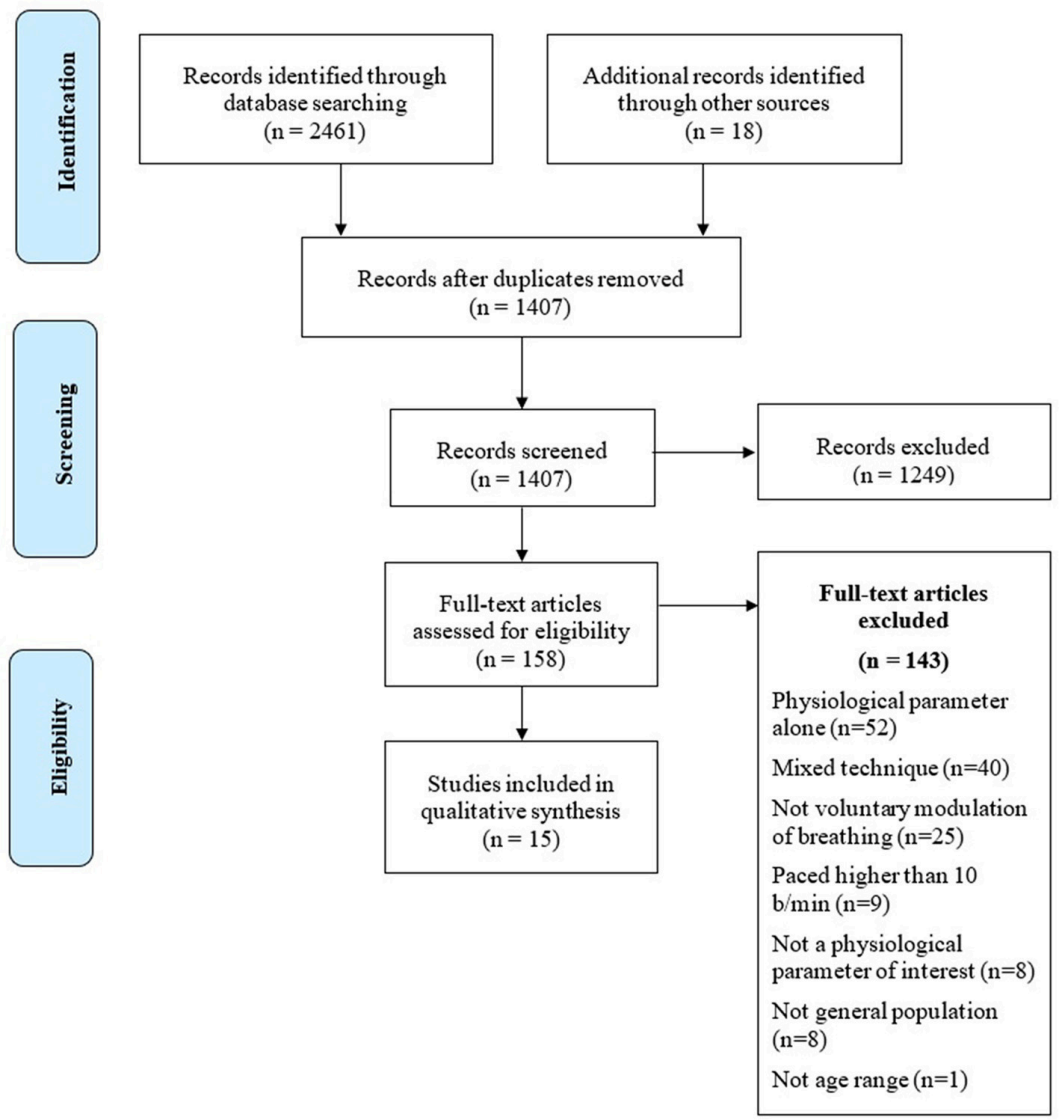
Flowchart of the study selection process.

The research of the studies, according to databases, terms and quantity of returned studies, is presented in Table 2. A complete flowchart of the study selection process is presented in Figure 1.

**Table 2:** Study research.

**Database**		**Query**	**Research in**	**Items found**
PubMed	#1	Prana OR Pranayama OR Pranayamic OR Pranayams OR “Breathing technique” OR “Breath technique” OR “Breathing exercise” OR “Breath exercise” OR “Paced breathing” OR “Paced breath” OR “Controlled breathing” OR “Controlled breath” OR “Slow breathing” OR “Slow breath” OR “Deep breathing” OR “Deep breath” OR “Metronome breathing” OR “Metronome breath” OR Yoga OR “Heart Rate Variability Biofeedback” OR “HRV Biofeedback”	Title/abstract	7,254
	#2	“Cardiorespiratory coherence” OR “Cardio respiratory coherence” OR “Cardio-respiratory coherence” OR “Cardiorespiratory coupling” OR “Cardio respiratory coupling” OR “Cardio-respiratory coupling” OR “Cardiorespiratory interaction” OR ”Cardio respiratory interaction“ OR ”Cardio-respiratory interaction“ OR ”Cardiorespiratory synchronization“ OR ”Cardio respiratory synchronization“ OR ”Cardio-respiratory synchronization“ OR Electroencephalogram OR EEG OR ”Functional connectivity“ OR ”Heart rate variability“ OR HRV OR ”Magnetic resonance imaging“ OR MRI OR ”Respiratory Sinus Arrhythmia“ OR RSA	Title/abstract	419,224
	#3	Combine #1 AND #2		997
	#4	Limit to ”Humans“		835
	#5	Limit to (English)		788
Scopus	#1	Prana OR Pranayama OR Pranayamic OR Pranayams OR ”Breathing technique“ OR ”Breath technique“ OR ”Breathing exercise“ OR ”Breath exercise“ OR ”Paced breathing“ OR ”Paced breath“ OR ”Controlled breathing“ OR ”Controlled breath“ OR ”Slow breathing“ OR ”Slow breath“ OR ”Deep breathing“ OR ”Deep breath“ OR ”Metronome breathing“ OR ”Metronome breath“ OR Yoga OR “Heart Rate Variability Biofeedback” OR “HRV Biofeedback”	Title/abstract/keywords	19,999
	#2	”Cardiorespiratory coherence“ OR ”Cardio respiratory coherence“ OR ”Cardio-respiratory coherence“ OR ”Cardiorespiratory coupling“ OR ”Cardio respiratory coupling“ OR ”Cardio-respiratory coupling“ OR ”Cardiorespiratory interaction” OR “Cardio respiratory interaction” OR “Cardio-respiratory interaction” OR “Cardiorespiratory synchronization” OR “Cardio respiratory synchronization” OR “Cardio-respiratory synchronization” OR Electroencephalogram OR EEG OR “Functional connectivity” OR “Heart rate variability” OR HRV OR “Magnetic resonance imaging” OR MRI OR “Respiratory Sinus Arrhythmia” OR RSA	Title/abstract/keywords	944,852
	#3	Combine #1 AND #2		1,897
	#4	Limit to (Humans OR Human)		1,772
	#5	Limit to (English)		1,673

### Study selection and characteristics

Two independent reviewers (AZ. and AP) checked an early pool of 2,461 abstracts collected from the search engines' outputs. Titles and abstracts were screened, and 2,303 studies were removed either because they were duplicated or of no interest for the systematic review. The remaining 158 full-text papers were checked for the eligibility criteria. At the end of the analysis, 15 articles meeting the eligibility criteria were retained and included in the review. Seven studies (Stark et al., 2000; Edmonds et al., 2009; Tsuji, 2010; Park and Park, 2012; Lin et al., 2014; Van Diest et al., 2014; Critchley et al., 2015) dealt with slow paced breathing. Five studies investigated the effects of HRV Biofeedback (Lehrer et al., 2003; Siepmann et al., 2008; Sakakibara et al., 2013; Gruzelier et al., 2014; Gross et al., 2016), two studies (Fumoto et al., 2004; Yu et al., 2011) analyzed the effects of Zen Tanden Breathing, and one (Kharya et al., 2014) investigated Prana-Yoga Breathing.

Descriptions of the methodologies employed in the included studies and their main results are presented in Tables 3, Tables 4, respectively, while details about physiological and psychological/behavioral data found in the studies are reported in Appendix 2.

**Table 3:** Included studies.

**Study**	**Study design**	**Slow breathing technique group**	**Control group**	**Mean age [control group]**	**Slow breathing technique**	**Slow breathing technique details**	**Comparison technique**	**Comparison technique details**
Critchley et al., 2015	Within subjects	20 (8 female)	No control group	34.5	Paced breathing (5.5 b/min; 10 b/min)	1 min each frequency	Spontaneous Breathing	1 min
Edmonds et al., 2009	Within subjects	14 (6 female)	No control group	33	Paced Breathing (6 b/min)	I/E = 1/1 + pause; 1/1 no pause; 1/2 + pause; 1/2 no pause	Spontaneous breathing	5 min
Fumoto et al., 2004	Within subjects	22 (6 female)	No control group	21–54 years	Voluntary abdominal breathing (Zen Tanden breathing) (3–4 b/min)	20 min	Spontaneous breathing	20 min
Gross et al., 2016	Within subjects	9 (6 female)	No control group	45.88	HRV biofeedback (6 b/min)	6 sessions (5 min with peacer + 5 min without pacer)	Spontaneous breathing	5 min
Gruzelier et al., 2014	Pre-post design	16	16	Unknown (I year bachelor students)	HRV biofeedback (6 b/min)	10 sessions (20 min each)	Non-intervention control group	Non-intervention control group
Kharya et al., 2014	Pre-post design	20 (10 female)	20 (Sudarshan Kriya) (10 female) 20 (Control) (10 female)	18–30 years [18–30 years]	Prana-Yoga (2–5 b/min)	150 days; 5 days a week; 30 min each session, nostril and mouth	Sudarshan Kriya (3–60 b/min) Spontaneous Breathing (Leisure Walking)	150 days; 5 days a week 30 min each session
Lehrer et al., 2003	Pre-post design	23 (16 female)	31 (22 female)	30.55 [27.93]	HRV Biofeedback (5.4–6 b/min)	10 days; 30 min, mouth	Spontaneous Breathing	10 days; 30 min each session
Lin et al., 2014	Within subjects	47 (36 female)	No control group	20.98	Paced breathing (5.5, 6 b/min)	2 min each frequency	Spontaneous breathing	5 min pre + 5 min post
Park and Park, 2012	Within subjects	58 (22 female)	No control group	Male: 24.8; Female: 24.5	Paced breathing (10 b/min)	15 min. I = 2.4 s; E = 3.6 s, nostril	Spontaneous breathing	15 min
Sakakibara et al., 2013	Pre-post design	15 (9 female)	15 (11 female)	22.8	HRV Biofeedback (6 b/min)	3 sessions, 20 min	Non-intervention control group	Non-intervention control group
Siepmann et al., 2008	Pre-post design	12 (6 female)	12 (6 female)	28	HRV Biofeedback (6 b/min)	6 sessions, 25 min	Non-intervention control group	Non-intervention control group
Stark et al., 2000	Within subjects	40 (20 female)	No control group	24.33	Paced breathing (9 b/min)	5 min	Paced breathing (12, 15, 18 b/min) Spontaneous breathing	5 min each frequency
Tsuji, 2010	Within subjects	10 (0 female)	No control group	21.7	Paced breathing (4 b/min)	10 min; I = 5 s, E = 10 s, nostril	Spontaneous breathing	10 min
Van Diest et al., 2014	Within subjects	23 (n. female non-specified)	No control group	1–22 years	Paced breathing (6 b/min)	I/E = 3/7, 7/3; 45 s each I/E ratio, nostril	Paced breathing (12 b/min) spontaneous breathing	7 min
Yu et al., 2011	Within subjects	15 (1 female)	No control group	38	Zen Tanden Breathing (3–4 b/min)	20 min; I = 6–8 s; E = 9–12 s	Spontaneous breathing	5 min pre + 5 min post

**Table 4:** Outcomes.

**Study**	**Cardio-respiratory system**	**Central nervous system**	**Time of physiological assessment**	**Psychological/Behavioral outcome**
Critchley et al., 2015	HRV anticorrelated with BOLD signal in anterior insula, DLPFC, left occipital cortex	Increased BOLD activity in pons, thalamus, cerebellum, striatum, subparabrachial nucleus, parabrachial nuclei, locus coeruleus, periaqueductal gray, hypothalamus, hippocampus, motor, supplementary motor and parietal cortices	During slow breathing technique	A trend for increased alertness Non-significant comfort changes (*ad-hoc* scales)
Edmonds et al., 2009	Increased LF, SDNN, pNN50 Decreased HF, VLF	Not investigated	During slow breathing technique	Increased ease and comfort (*ad-hoc* scales)
Fumoto et al., 2004	Not investigated	Increased high-frequency alpha power	During and immediately after slow breathing technique	Increased vigor-activity (Profile of Mood States) Non-significant anxiety changes (State Trait Anxiety Inventory)
Gross et al., 2016	Increased HRV, SDNN, LF	Not investigated	During slow breathing technique	Increased somatic emotional regulation strategies (Somatic Strategies and Somatic Suppression scale)
Gruzelier et al., 2014	Increased SDNN	Not investigated	During slow breathing technique	Decreased anxiety (Depression, Anxiety, and Stress Scale)
Kharya et al., 2014	Non-significant change HRV, HF, LF/HF	Not investigated	After long-term slow breathing technique intervention (during rest)	Increased lifestyle management (Self “Well-Being” Inventory and Depression Screening Test)
Lehrer et al., 2003	Increased HRV, LF Decreased HF	Not investigated	During and immediately after slow breathing technique	Non-significant relaxation changes (Relaxation Inventory) Decreased side-effects of relaxation training (Side Effects of Relaxation Scale)
Lin et al., 2014	Increased LF, LF/HF, SDNN Non-significant change HF	Not investigated	During slow breathing technique	Increased relaxation Non-significant anxiety changes (*ad-hoc* scales)
Park and Park, 2012	Increased HF Decreased LF/HF Non-significant LF change	Increased alpha power Decreased theta power	During slow breathing technique	HF anticorrelated with cooperativeness and self-transcendence LF anticorrelated with self-transcendence Alpha power correlated with harm avoidance, novelty seeking, persistence, self-directedness, self-transcendence (Temperament and Character Inventory)
Sakakibara et al., 2013	Increased HF	Not investigated	Immediately after (during sleep in the following night)	Non-significant anxiety changes (State Trait Anxiety Inventory)
Siepmann et al., 2008	Non-significant change HRV	Not investigated	After long-term slow breathing technique intervention (during rest)	Non-significant mood changes (Beck Depression Inventory)
Stark et al., 2000	Increased HRV, LF, HF, LF/HF	Not investigated	During slow breathing technique	Non-significant emotional state changes (Self-Assessment Manikin Scale)
Tsuji, 2010	Not investigated	Non-significant change alpha power	During and immediately after slow breathing technique	Non-significant changes in mood (Two-Dimensional Mood Scale)
Van Diest et al., 2014	Increased LF Decreased HF Increased RSA	Not investigated	During slow breathing technique	Higher positive energy, higher pleasantness, and lower arousal
Yu et al., 2011	Not investigated	Increased alpha power Decreased theta power Increased oxygenated hemoglobin in anterior Prefrontal cortex	During and immediately after slow breathing technique	Decreased tension–anxiety, depression–dejection, anger–hostility, confusion (Profile of Mood States)

### Synthesized findings

#### Breath and the cardio-respiratory system

##### Slow paced breathing and the cardio-respiratory system

An association between cardio-respiratory parameters and psychological/behavioral outcomes related to slow paced breathing was found coherently in four studies. Edmonds et al. (2009) showed that paced breathing sessions at 6 b/min with different inspiration/expiration ratios increased the Standard deviation of all NN intervals (SDNN) and HRV in the Low Frequency (LF) range, while reducing contributions both in the High Frequency (HF) and in the Very Low Frequency (VLF) ranges. A relationship was found between physiological variables and psychological/behavioral outcomes: using single-item scales, participants reported the strongest perceived ease and comfort level in association with the breathing condition characterized by the highest SDNN and LF values. Park and Park (2012) found an increase of HF power paralleled by a decrease in LF/HF ratio during paced breathing at 10 b/min as compared to spontaneous breathing. No significant difference between the two conditions was observed when considering LF power. Personality traits were evaluated using the Temperament and Character Inventory (Lee and Hwang, 2009). Cooperativeness showed an inverse correlation with HF power, while Self-Transcendence was inversely correlated with both LF and HF power. Lin et al. (2014), during paced breathing at 6 and 5.5 b/min with two different inspiration/expiration ratios (5:5 and 4:6), found higher SDNN, LF power and LF/HF ratio, and no significant differences in HF power coherently for all paced breathing sessions as compared to the control condition (spontaneous breathing). All paced breathing sessions were associated with an increased subjective perception of relaxation as compared to the control condition; at variance, no difference in subjectively perceived anxiety was found between paced breathing and control sessions. Van Diest et al. (2014) observed higher RSA, higher LF and lower HF power during 6 b/min paced breathing with different inspiration/expiration ratios, as compared to 12 b/min. Paced breathing at 6 b/min was characterized, at a subjective level, by higher positive energy, higher pleasantness, and lower arousal levels, as measured with the Smith Relaxation States Survey (Smith, 2001), when compared to 12 b/min breathing.

Only two studies found no clear association between cardio-respiratory parameters and psychological/behavioral outcomes related to slow paced breathing. Stark et al. (2000) found that paced breathing at 9 b/min was associated with higher HRV, LF, and HF power, and higher LF/HF ratio, as compared with higher paced breathing frequencies (12, 15, and 18 b/min). However, no difference in emotional scores of the Self-Assessment Manikin Scale (Bradley and Lang, 1994) and in a single-item mental effort measure was found among these different paced breathing frequencies. Kharya et al. (2014) found no difference in HF and LF power, and LF/HF ratios between Prana-Yoga (slow breathing), Sudarshan Kriya Yoga (fast breathing), and control condition (spontaneous breathing), after 150 days of practice (5 days a week/30 min a day). On the psychological/behavioral side, an improvement in the Life Style Management Scale was found in Prana-Yoga group as compared to controls.

##### HRV biofeedback and the cardio-respiratory system

An association between cardio-respiratory parameters and psychological/behavioral outcomes related to HRV Biofeedback was found in three studies. Lehrer et al. (2003) found that 10 sessions of biofeedback (keeping the breathing frequency in the 5.4 b/min-to-6 b/min range for 30 min) induced an increase in HRV and LF power, and a concurrent decrease in HF power, as compared to the control condition (spontaneous breathing). It is important to highlight that high HRV total power was maintained during a post-session resting-state period, during which respiratory frequency returned to normal. Moreover, indicating a cumulative effect of Biofeedback training, HRV was significantly higher at the end of each session (the last 5 min) than at the beginning (the first 5 min). Subjects after the biofeedback session reported significantly lower adverse effects, as measured by the Side Effects of Relaxation Scale (Kotsen et al., 1994) (e.g., anxiety, intrusive thoughts, or fear of losing control), but no effects on relaxation, as measured with the Relaxation Inventory (Crist et al., 1989). Gross et al. (2016) found that 5 sessions of HRV Biofeedback increased total HRV (HRV total power and SDNN) and LF power in members of the support and management staff of elite sport environment, compared to baseline. At the psychological/behavioral level there were no changes in lifestyle variables, and in emotional regulation based on cognitive reappraisal and expression suppression [as measured with the Emotion Regulation Questionnaire (Gross and John, 2003)]. However, authors found increased habitual use of adaptive, somatic-based, emotional regulation strategies after HRV Biofeedback interventions (as measured with the Somatic Strategies and Somatic Suppression scale, Gross et al., 2016). Gruzelier et al. (2014) investigated the effects of 10 sessions of HRV Biofeedback on dance conservatoire students, compared to a no-intervention group. They found a significant increase in SDNN only in the HRV Biofeedback group. At the psychological/behavioral level, anxiety levels (assessed with the Depression, Anxiety, and Stress Scale, Lovibond and Lovibond, 1995) decreased in the HRV Biofeedback group as compared with the control group. There was no difference in the other psychological variables assessed (i.e., creativity, with the Insight Problems and the Alternate Uses Tests).

Two studies found no clear association between cardio-respiratory parameters and psychological/behavioral outcomes related to HRV Biofeedback. Sakakibara et al. (2013) compared the effects of HRV Biofeedback, autogenic training, and no-treatment control on healthy young adults, practiced before bedtime, on HRV during the two following nights. They found that HF power increased during sleep only in the Biofeedback group, whereas it did not change in the autogenic training and in control groups. Moreover, HF power was higher during both nights in the HRV Biofeedback group, compared to autogenic training and control groups. However, authors found no differences in state anxiety (measured before bedtime with the State-Trait Anxiety Inventory, Spielberger et al., 1983) between the three groups. Siepmann et al. (2008) enrolled both depressed and healthy subjects, who attended 6 sessions of HRV biofeedback, and were compared with healthy subjects during an active control condition. No significant changes of HRV were observed in healthy subjects after HRV Biofeedback sessions. Moreover, no psychological/behavioral changes, as measured with the Beck Depression Inventory (Beck et al., 1961) and the State-Trait Anxiety Inventory, were registered.

#### Breath and central nervous system

Four studies consistently found an association between neurophysiological parameters and psychological/behavioral outcomes. Fumoto et al. (2004) found that voluntary abdominal breathing (Zen Tanden Breathing) at 3–4 b/min significantly reduced alpha peak at 10 Hz at the EEG and induced significantly higher alpha2 activity (10–13 Hz) in the parietal areas as compared to spontaneous breathing. At a subjective level, participants reported improved vigor-activity in the Profile of Mood States (McNair et al., 1971) subscale scores, and reduced anxiety, evaluated with both Profile of Mood States subscale and State-Trait Anxiety Inventory (Spielberger et al., 1983) (even if the between-condition score difference was not significant). Yu et al. (2011), during Zen Tanden Breathing at 3–4 b/min, found significantly increased level of oxygenated hemoglobin, as measured by Near-Infrared Spectroscopy, in the anterior part of the prefrontal cortex (Brodmann area 9 and 10), paralleled by an increase in EEG alpha band activity, and a decrease in theta band with respect to spontaneous breathing. After Zen Tanden Breathing, subjects reported reduced scores in Tension-Anxiety, Depression-Dejection, Anger-Hostility, and Confusion subscales of the Profile of Mood States as compared to the control condition. During paced breathing at 10 b/min, Park and Park (2012) found decreased EEG theta power on left frontal, right temporal and left parietal areas, and increased alpha power over the whole cortex as compared to spontaneous breathing. Personality traits such as Harm Avoidance, Novelty Seeking, Persistence, Self-Directedness, and Self-Transcendence (Temperament and Character Inventory subscales), positively correlated with EEG alpha power. Critchley et al. (2015), in a fMRI study, found increased Blood Oxygenation Level Dependent (BOLD) activity in a large number of brain areas during paced breathing at 5.5 b/min, as compared to 10 b/min. Sub-cortical structures included: (1) the dorsal length of the pons, (2) thalamic regions, (3) cerebellum, (4) striatum, (5) Kölliker-Fuse (sub-parabrachial nucleus), (6) parabrachial nuclei, (7) locus coeruleus, (8) periaqueductal gray, (9) hypothalamus, (10) hippocampus. Activated cortical areas were: (1) motor, (2) supplementary motor, and (3) parietal cortices. Across all participants, a trend for increased alertness (measured with a single-item visual analog scale) was found during 5.5 b/min condition when compared to the control condition. This is the only study included in this review that attempted a correlation between brain activity and HRV: authors found a positive correlation between HRV and activations of the medulla and hippocampus, and a negative one with activity in the anterior insula, dorsomedial prefrontal cortex and left occipital cortex.

Finally, Tsuji (2010) did not find any difference between slow (4 b/min) and spontaneous breathing either when considering EEG alpha power or mood self-assessment using the Two-Dimensional Mood Scale (Sakairi et al., 2013). A possible explanation of these negative findings could stem from the low statistical power of the study (only ten subjects were enrolled).

### Risk of bias

The vast majority of records checked were focused on the contribution of slow breathing techniques on the clinical outcomes of chronic and acute pathologies, and therefore were excluded from the review. Many studies investigated the effects of interventions characterized by a combination of breathing techniques, postures and meditation, while others investigated the effects of emotional stimulation while performing a specific breathing technique. As paced breathing was either intermingled with other kind of interventions or used during active stimulation of the subjects (e.g., anger or stress induction), all these studies were excluded from the review, as they did not allow the unambiguous identification of the specific psychophysiological effects of breath modulation. A large number of studies were excluded as they focused on techniques not aimed at a conscious regulation of breathing, requiring, on the contrary, the meditator not to attempt any control on his/her own breathing rhythm, but rather to observe it in a non-judgmental way. Finally, several other studies lacked a rigorous description of the experimental set-up and of the applied methodologies, impeding thus the study replicability, and were consequently excluded from the review (for reasons for the exclusions of all studies, see Figure 1).

As regards the included studies, 10 adopted within-subject designs, and 5 adopted pre-post designs. No studies adopted longitudinal or randomized controlled designs. Risk of bias and methodological quality of the included studies were assessed independently by the first two authors (AZ and AP), using two different tools. Disagreements between the reviewers were resolved by discussion with a third reviewer (AG). As regards within-subject designs, the Single-Case Reporting Guideline In Behavioural Interventions (SCRIBE) Statement (Tate et al., 2016a,b) was followed. As regards pre-post designs, a Quality Assessment Tool adapted from several published systematic reviews (see Cummings et al., 2008) was adopted. Both assessment tools revealed that the quality of the included studies ranged from sufficient to good. Regarding within-subjects designs, the main concerns were related to the absence of any blinding condition (which intrinsically depends on the slow breathing techniques interventions), lack of description of participants demographic data, and missing access to raw databases and to protocol designs. Regarding pre-post designs, the main concerns relate to sampling methods, sample sizes non-statistically justified, and lacking of randomization in group assignment. Check-lists are presented for within-subjects and for pre-post designs in Supplementary Tables 2, 3, respectively.

## Discussion

### Summary of main findings

We have herein reviewed the literature on the psychophysiological effects of both eastern and western slow breathing techniques with the aim of identifying the physiological mediators at the basis of their demonstrated psychological and behavioral beneficial effects. We found interesting albeit limited evidence of a relationship between physiological parameters and psychological/behavioral outcomes in healthy subjects undergoing slow breathing techniques. We must underline that the paucity of collected evidence is mostly ascribable to the heterogeneity of the investigated techniques and of the participants selection criteria. Consequently, in some cases, results stemming from different studies lead to contradictory conclusions (see Table 4). Moreover, no study explicitly estimated the correlations between physiological modifications and psychological/behavioral outcomes, with the notable exception of Park and Park (2012), which, however, focused on the correlation between changes of HRV- and EEG-related physiology (during slow breathing techniques) and stable personality traits, and not on psychological/behavioral state changes directly related to slow breathing techniques. In spite of these limitations, we identified some common trends when considering specific cardio-respiratory and central nervous system parameters on the one side, and positive psychological/behavioral outcomes on the other.

Slow breathing techniques (related both to slow paced breathing and to HRV Biofeedback) seem to interact with the cardio-respiratory system by increasing HRV and RSA, suggesting thus a strong involvement of the parasympathetic nervous system (Reyes del Paso et al., 1993; Berntson et al., 1997). At variance, when considering HF and LF power, a heterogeneous and contradictory set of outcomes was found, mainly depending on the breathing frequency: Park and Park (2012), and Stark et al. (2000) observed HF power increases (slow breathing techniques vs. control condition), while other studies found no changes (Siepmann et al., 2008; Kharya et al., 2014; Lin et al., 2014) or even HF power decreases (Lehrer et al., 2003). Moreover, Sakakibara et al. (2013) found that an HRV Biofeedback session before sleep increased HF during sleep in the following night. When considering LF power, a group of studies highlighted increases in the slow breathing techniques-control comparison (Stark et al., 2000; Lehrer et al., 2003; Edmonds et al., 2009; Lin et al., 2014; Van Diest et al., 2014; Gross et al., 2016), while other authors found no difference between the two conditions (Park and Park, 2012; Kharya et al., 2014).

Despite these contradictions, a common trend emerges in some of the included studies, namely the association between the increase of HRV-SDNN power and of LF power during slow breathing techniques (at near 6 b/min) and psychological/behavioral outcomes of decreased anxiety (Gruzelier et al., 2014), side effects of relaxation (Lehrer et al., 2003), and arousal (Van Diest et al., 2014), together with increased ease and comfort (Edmonds et al., 2009), relaxation (Lin et al., 2014), positive energy and pleasantness (Van Diest et al., 2014) and, interestingly, somatic-based emotional control strategies (Gross et al., 2016). We hypothesize that increased HRV and LF power could be an important physiological substrate related to psychological/behavioral positive outcomes of slow breathing techniques. However, it is important to stress the fact that abovementioned studies did not measure HRV features immediately after the session, but during the slow breathing techniques (with the notable exception of Lehrer et al., 2003). This can be a confounding factor, because slow breathing at 6 b/min can amplify oscillations at the breath frequency in the LF power band (Aysin and Aysin, 2006). However, the study from Lehrer et al. (2003) provides evidence that HRV power can stay high during a post-session resting-state period, during which respiratory frequency returns to normal.

When considering the central nervous system, slow breathing techniques were often paralleled by increases of alpha and decreases of theta power (Fumoto et al., 2004; Yu et al., 2011; Park and Park, 2012), when considering scalp EEG activity, a finding that may reflect the brain “idle” state at rest (Ben-Simon et al., 2008) and the synchronization in the Default Mode Network (DMN) (Knyazev et al., 2011). Measured with by Near-Infrared Spectroscopy, Yu et al. (2011) reported increased levels of oxygenated hemoglobin in the anterior part of the prefrontal cortex. Moreover, in the only fMRI study (Critchley et al., 2015), slow breathing techniques were found to increase BOLD activity in the prefrontal, motor, and parietal cortices, areas related to voluntary breathing, as well as in sub-cortical areas as the pons, the thalamus, the sub-parabrachial nucleus, the periaqueductal gray, and the hypothalamus, areas involved also in the regulation of internal bodily states. The authors found also that insular activation anti-correlated with HRV power. The modulation of central nervous system activity by slow breathing techniques, resulting in increase of EEG alpha power and decrease of EEG theta power was reliably found to be associated with positive outcomes, improving vigor-activity, and reducing anxiety, depression, anger and confusion when considering psychological/behavioral outcomes (Fumoto et al., 2004; Yu et al., 2011).

Starting from the results reported in this systematic review, the construction of a psychophysiological model of slow breathing techniques can be attempted. In general, slow breathing techniques enhance interactions between autonomic, cerebral and psychological flexibility, linking parasympathetic and CNS activities related to both emotional control and well-being. Slow breathing techniques seem to promote a predominance of the parasympathetic autonomic system with respect to the sympathetic one, mediated by the vagal activity (Streeter et al., 2012; Brown et al., 2013). The vagus nerve in turn, transmits interoceptive information from gastrointestinal, cardiovascular and pulmonary systems to the central nervous system through the Nucleus of the Tractus Solitarius. The enhancement of vagal tone within the cardiovascular system is reflected by the increase of both HRV power and RSA. It is worth underlining that HRV modulation is highly dependent on the respiration frequency, increasing along with the slowing of breath (Song and Lehrer, 2003). RSA on its side is consistently considered a robust index of parasympathetic activity (Reyes del Paso et al., 1993), and it has proven to be mainly driven by two mechanisms: (1) the decrease of intrathoracic pressure during inhalation that promotes an increase of venous return, which in turn is registered by stretch receptors causing increases in heart rate (Bainbridge Reflex, Bainbridge, 1915), and (2) the inhibition of vagal cardiac efferent activity due to the stimulation of pulmonary C-fiber afferents (Shykoff et al., 1991; Horner et al., 1995; De Burgh Daly, 2011). There is growing evidence suggesting an active role of RSA in regulating homeostasis and improving oxygen uptake (Hayano et al., 1996; Yasuma and Hayano, 2004) and pulmonary gas exchange during slow breathing techniques (Bernardi et al., 1998; Giardino et al., 2003). In this framework, we found consistent proofs linking the slowing of breath rhythm to increases in RSA (Van Diest et al., 2014). Jerath et al. (2006) hypothesized another slow breathing techniques-related mechanism, which would explain the parasympathetic nervous system activity predominance. He hypothesized an involvement of lungs stretch receptors (i.e., Herin Breuer's reflex) and of stretching pulmonary connective tissue (fibroblasts). The stretching of lung tissue in fact produces inhibitory signals, as the fibroblasts activity fosters a slow adaptation of stretch receptors and hyperpolarization currents (Matsumoto et al., 2000; Kamkin et al., 2005).

Slow breathing techniques at 9–10 b/min, is usually associated with HF power increase (Stark et al., 2000; Park and Park, 2012): of note, HF power is usually considered an index of parasympathetic activation (Task Force of the European Society of Cardiology the North American Society of Pacing Electrophysiology, 1996). On the contrary, slower breathing (at around 6 b/min) increases LF power (Stark et al., 2000; Lehrer et al., 2003; Edmonds et al., 2009; Lin et al., 2014; Van Diest et al., 2014), and is usually associated with sympathetic activation (Vincent et al., 2008). However, as already mentioned, the interpretation of these results is not so straightforward since very low respiratory frequencies overlap the frequency interval of LF power (0.04–0.15 Hz), possibly causing a “false-positive” increase of power (Aysin and Aysin, 2006).

Subsequently, the shift toward a parasympathetic predominance is conveyed to the central nervous system via the Nucleus of the Tractus Solitarius, which sends its projection to the thalamus and limbic system via the parabrachial nucleus (Streeter et al., 2012; Brown et al., 2013). In this framework, Critchley et al. (2015) found an anti-correlation between insular BOLD activity and HRV during slow breathing techniques.

At the same time, slow breathing techniques are necessarily driven by brain top-down processes stemming from the voluntary shift of attention toward breath monitoring aiming at the active control of breathing rhythm. The nature of these top-down processes could be inferred from the model developed by Gard et al. (2014) for yoga, which, while being a more complex discipline involving physical and mental practices, shares some notable commonalities with slow breathing techniques. Gard's model hypothesizes that yoga may involve top-down components such as attention, working memory, and executive monitoring. Brain networks associated with these functions are the central executive network, including both the dorsolateral prefrontal and the posterior parietal cortices (Goulden et al., 2014), and the fronto-parietal network, including the dorsolateral prefrontal and the anterior cingulate cortices, the inferior frontal junction, the pre-supplementary motor area, and the intraparietal sulcus (Seeley et al., 2007; Vincent et al., 2008; Harding et al., 2015). Taylor et al. (2010) in a review about mind-body therapies (i.e., techniques focusing on functional links between mind and body) such as slow breathing techniques, suggested the existence of an executive homeostatic network as a fundamental substrate of these practices. This network includes the anterior cingulate, the prefrontal and the insular cortices, areas involved in physiological self-awareness and cognitive modulation. This hypothesis is partially supported by Critchley et al. (2015) and Yu et al. (2011), who found BOLD activations in the anterior prefrontal, motor, supplementary motor and parietal cortices during slow breathing techniques.

At the EEG level, slow breathing techniques are associated with reductions in theta and increases in alpha activity. The increase of alpha power is in line with the results described in a recent systematic review dealing with the neurophysiology of mindfulness (Lomas et al., 2015), and has been interpreted as an index of an increased inwardly directed attention (i.e., to the self-regulated act of breathing). We hypothesized that the progressive sensory deafferentation occurring during slow breathing techniques induces an inward directed attentional shift allowing both alpha increase and higher DMN synchronization. The thalamus, strongly engaged in a burst mode activity in the alpha range, impedes the expression of other pacemakers such those underlying theta rhythms. According to this hypothesis, the deepening of meditative state allows the emergence of theta rhythm which owing to its off-periods, plays a fundamental role in altering the state of consciousness.

Unexpectedly, the majority of slow breathing techniques studies did not directly investigate slow breathing techniques effects on the state of consciousness, even if its modification is considered one of the mail goals of Pranayama (Iyengar, 1985). To our best knowledge, only one study analyzed breath-related alterations of the state of consciousness, but it adopted a fast breathing technique (Holotropic Breathwork, Rock et al., 2015). We speculate that the subjective experience of an altered state of consciousness depends on the rearrangement of cortical functional connectivity, in particular within the DMN, a set of cortical structures whose activity was found to be associated with altered states of consciousness induced by meditation (Brewer et al., 2011), by psychedelic substances (Carhart-Harris et al., 2014), and by sleep (Chow et al., 2013).

Another neurophysiological framework explaining the link between slow breathing techniques and consciousness is related to the fine-tuning of thalamic and cortical activities exerted by the olfactory bulb. The neural patterns of this structure are modulated by the mechanical stimulation of the olfactory epithelium during nostril breathing (Fontanini and Bower, 2006; Piarulli et al., 2018). Even if not specified in all studies (see Table 3), it is plausible that the majority of slow breathing techniques are performed via nasal respiration (Jerath et al., 2006). Moreover, as historically noted (Ramacharaka, 1903), nostril breathing is a fundamental aspect of every form of meditation. Studies on the animal model, as well as on specific Pranayama techniques, suggest that nasal breathing is able to modulate both the autonomic system and brain activity through receptors located in the superior nasal meatus, which are sensitive both to mechanical and chemical stimuli (Wrobel and Leopold, 2005; Buonviso et al., 2006; Kepecs et al., 2006). Early studies both on the animal model and humans found a direct relationship between nasal stimulation and brain activity, independent from thoracic respiratory activity, which was abolished by anesthesia of the nasal mucosa (Adrian, 1942; Hobson, 1967; Servít and Strejckovà, 1976; Servit and Strejckovà, 1979; Kristof et al., 1981; Servít et al., 1981; Sobel et al., 1998). More recently, other studies demonstrated the presence of significant oscillations at the same frequency of the respiratory rate in a number of brain cortical and subcortical areas, which disappeared after tracheotomy, and were restored, independently from thoracic respiration, by the rhythmic delivery of air-puffs into the nasal cavity. These areas included the olfactory bulb, the piriform cortex, the somatosensory cortex, the prefrontal cortex, and the hippocampus (Fontanini et al., 2003; Ito et al., 2014; Viczko et al., 2014; Yanovsky et al., 2014; Lockmann et al., 2016; Nguyen Chi et al., 2016; Biskamp et al., 2017; Liu et al., 2017; Wu et al., 2017; Zhong et al., 2017). The modulating effect of nostril breathing on the activity of the piriform cortex, amygdala and hippocampus has been unambiguously demonstrated in humans (Zelano et al., 2016).

Based on these evidence, a recently published study from our laboratory (Piarulli et al., 2018) found that ultra-slow mechanical stimulation of olfactory epithelium induced an enhancement of delta-theta EEG activity over the whole cortex, mainly involving DMN structures, associated to a reversal of the overall information flow directionality from postero-anterior to antero-posterior, and to an altered state of consciousness, phenomenologically overlapping those experienced in deep meditative states.

Taken together, these results confirm that nasal stimulation represents the fundamental link between slow breathing techniques, brain and autonomic activities and psychological/behavioral outputs. Future studies should be aimed at verifying this hypothesis, possibly comparing brain activity during slow respiration when performing nasal breathing with that detected during mouth breathing.

### Limitations

A general consideration emerging from this systematic review is the lack in scientific literature of a standardized methodology, both when considering the experimental design and the description of breathing techniques. The initial aim of this work was to conduct a meta-analysis of the existent literature, but due to the heterogeneity of the selected experimental groups, of the interventions, and of the outcomes, a statistical pooling was infeasible. This issue was already highlighted in Gotink et al. (2016) and in Posadzki et al. (2015) when dealing with yoga and mindfulness-based interventions, respectively. As an indication for future research, future research will have to: (i) directly disentangle the role of each aspect of breathing and meditation practices; (ii) measure both physiological and psychological/behavioral variables, in order to draw correlations and (possibly) causal connections between slow breathing techniques and health; (iii) investigate long-term effects of slow breathing techniques practice, adopting more robust longitudinal studies; and (iv) consider the possibility of adverse effects of slow breathing techniques.

Moreover, in order to increase methodological quality in breathing technique's research, we propose a checklist their precise description in scientific literature. Nash and Newberg (2013) have recently stated the importance of breath in every meditation technique. In their attempt to create a taxonomy for meditation, breath is the eighth point that must be described for a scientific definition of a meditation technique. However, they suggest to state only “whether there are any specific recommendations for type or control of breathing.” In order to promote a more standardized research on breathing techniques, we propose to adopt an expanded checklist, as it follows:

- Specifying whether breath is consciously attended or not
- Specify if other techniques are associated with breathing (e.g., “feeling the breath in the body,” sounds with mouth, breath-related mantras, breath-related imagery, etc.)
- Specify the mean breathing frequency and, if present, any significant breathing frequency variations
- Specify whether during respiration the air passes through the mouth or through the nostrils (both, left, right, alternate), or through both mouth and nostrils
- Specify the presence and the duration of inspiration (if any) and expiration pauses (if any)
- Specify the Inspiration/Expiration ratio
- Specify whether the breath is thoracic or abdominal
- Specify (if applicable) what type of metronome is used
- Specify (if applicable) the air pressure during the inspiratory phases.

### Conclusions

We found evidence of increased psychophysiological flexibility linking parasympathetic activity, CNS activities related to emotional control and psychological well-being in healthy subjects during slow breathing techniques. In particular, we found reliable associations between increase of HRV power and of LF power, increase of EEG alpha and decrease of EEG theta power, induced by slow breathing techniques at 6 b/min, and positive psychological/behavioral effects. This evidence is unfortunately weakened by the lack of clear methodological descriptions that often characterizes slow breathing techniques literature. Further studies are thus needed to unambiguously assess these links. Only few authors have attempted to systematically describe the psychophysiological effects of slow breathing techniques, and a fewer number have attempted to relate them to meditation practice. Breath seems to be confined to an “ancillary” role when compared to other important mechanisms such as cognitive or affective ones.

Finally, more research is needed to disentangle the pure contribution of breathing in a variety of meditation techniques. As stated by Nash and Newberg (2013), different methods (e.g., attentional-based and breath-based techniques) could lead to similar states. We herein proposed a brief check-list that could help to improve research on this topic. In our opinion, it is possible that certain meditative practices and slow breathing techniques share, up to a point, similar mechanisms. Some converging data exist regarding the mutual relationships between HRV, RSA, theta, and alpha EEG bands activity, the activation of cortical and sub-cortical brain areas, and positive psychological/behavioral outcomes. In addition, the role that nostrils (and more specifically, the olfactory epithelium) play during slow breathing techniques is not yet well considered nor understood: evidence both from animal models and humans support the hypothesis that a nostril-based respiration stimulating the mechanoceptive properties of olfactory epithelium, could be one of the pivotal neurophysiological mechanisms subtending slow breathing techniques psychophysiological effects.

## Author contributions

AG, EG, and AZ conceived the idea. AZ and AP conducted the literature search and analysis. AZ, AP, EG, and AG wrote the paper. ML, DM, and BN contributed to the writing. All authors reviewed the manuscript.

### Conflict of interest statement

The authors declare that the research was conducted in the absence of any commercial or financial relationships that could be construed as a potential conflict of interest.

## Abbreviations

DMN: Default Mode Network.
