# Supplementary material for: How Breath-Control Can Change Your Life: A Systematic Review on Psycho-Physiological Correlates of Slow Breathing
Source: Front Hum Neurosci. 2018 Sep 7;12:353. doi: 10.3389/fnhum.2018.00353 (PMC6137615; doi:10.3389/fnhum.2018.00353)
Supplement: Supplementary file 1 [file Table_1.DOCX]

Supplementary Material

How breath-control can change your life: a systematic review on psycho-physiological correlates of slow breathing

**Andrea Zaccaro^1^, Andrea Piarulli^1,2^, Marco Laurino^3^, Erika Garbella^4^, Danilo Menicucci^1^, Bruno Neri^5^, Angelo Gemignani^1,3,6^***

^1^ University of Pisa, Department of Surgical, Medical, Molecular and Critical Area Pathology, Pisa, Italy

^2^ Coma Science Group, GIGA Consciousness, University of Liège, Liège, Belgium

^3^ National Research Council, Institute of Clinical Physiology, Pisa, Italy

^4^ Nuovo Ospedale degli Infermi, Biella, Italy

^5^ University of Pisa, Department of Information Engineering, Pisa, Italy

^6^ Azienda Ospedaliero-Universitaria Pisana, Pisa, Italy

*** Correspondence:**Angelo Gemignani
angelo.gemignani@unipi.it

**Appendix 1: Keywords**

**Breathing Techniques**

Breath Exercise

Breath Technique

Breathing Exercise

Breathing Technique

Controlled Breath

Controlled Breathing

Deep Breath

Deep Breathing

Metronome Breath

Metronome Breathing

Paced Breath

Paced Breathing

Prana

Pranayam

Pranayama

Pranayamic

Pranayams

Slow Breath

Slow Breathing

Heart Rate Variability Biofeedback

HRV Biofeedback

**Outcomes**

Cardio respiratory Coherence

Cardio respiratory Coupling

Cardio respiratory Interaction

Cardio respiratory Synchronization

Cardiorespiratory Coherence

Cardio-respiratory Coherence

Cardiorespiratory Coupling

Cardio-respiratory Coupling

Cardiorespiratory Interaction

Cardio-respiratory Interaction

Cardiorespiratory Synchronization

Cardio-respiratory Synchronization

EEG

Electroencephalogram

Functional Connectivity

Heart Rate Variability

HRV

Magnetic Resonance Imaging

MRI

Respiratory Sinus Arrhythmia

RSA

**Appendix 2: Parameters/Psychometric questionnaires discussed in the review**

**Physiological parameters discussed in the review**

HRV total power (ms^2^): Heart Rate Variability, total power of the variance of normal-to-normal (NN) heartbeat intervals over a temporal segment (frequency approximately ≤ 0.4 Hz).

VLF (ms^2^): Power in very low frequency range (frequency < 0.04 Hz). Controversial role: related to vagal tone, thermoregulation, and renin-angiotensin-aldosterone system.

LF (ms^2^): Power in low frequency range (frequency 0.04-0.15 Hz). It reflects baroreceptor-mediated sympathetic and parasympathetic influences on heart rate.

HF (ms^2^): Power in high frequency range (frequency 0.15-0.4 Hz). It is a marker of parasympathetic activation. It reflects also respiratory influences on the heart rate.

LF/HF: Ratio LF [ms2]/HF [ms2]. A measure of sympatho-vagal balance. Its decrease is a marker of parasympathetic activity.

SDNN (ms): Standard deviation of all NN intervals. It measures the HRV total power in the time domain.

RSA (ms): Respiratory Sinus Arrhythmia, difference between maximum and minimum cardiac interbeat interval per breath. It is a measure of parasympathetic nervous system activity.

**Psychometric questionnaires discussed in the review**

Profile of Mood States (McNair et al., 1971): it is a self-administered questionnaire used to assess the mood state. It consists of 65 adjectives on a 5-point Likert scale (0 = not at all; 5 = extremely). It is divided in the following subscales: Tension-Anxiety, Depression-Dejection, Anger-Hostility, Fatigue-Inertia, Vigor-Activity, Confusion-Bewilderment.

State-Trait Anxiety Inventory (Spielberger et al., 1983): it is a self-administered questionnaire used to assess trait and state anxiety. It consists of 20 items for trait anxiety and 20 items for state anxiety on a 4-points Likert scale (1 = almost never; 4 = almost always).

Temperament and Character Inventory-Revised short version (Lee and Hwang, 2009): it is a self-administered questionnaire for personality traits. It consists of 140 items on a 5-point Likert scale (0 = totally disagree; 5 = totally agree). It is divided in four subscales assessing temperaments (Novelty Seeking, Harm Avoidance, Reward Dependence, and Persistence) and three subscales assessing characters (Self-Directedness, Cooperativeness, and Self-Transcendence).

Self-Assessment Manikin Scale (Bradley and Lang, 1994): it is a non-verbal pictorial self-assessment technique for measure the affective reactions to a wide variety of stimuli and situations. It measures Pleasure, Arousal, and Dominance of the emotional responses.

Smith Relaxation States Survey (Smith, 2001): it is a self-administered questionnaire used to measure the experiences people have during different kinds of relaxation. It consists of 38 items on a 6-point Likert scale (1 = not at all; 6 = the maximum). It is divided in the following subscales: Basic Relaxation, Mindfulness, Positive Energy, Transcendence, and Stress Relaxation Inventory (Crist et al., 1989): it is a self-administered scale used to measure three dimensions of the relaxation experience. It consists in 45 items on a 5-point Likert scale. It is divided in the following subscales: Physiological Tension, Physical Assessment, and Cognitive Tension.

Side Effects of Relaxation Scale (Kotsen et al., 1994): it is a self-administered scale used to measure common adverse experiences during relaxation training.

Two-Dimensional Mood Scale (Sakairi et al., 2013): it is a self-administered scale used to measure mood. It consists in 8 items on a 6-point Likert scale (0 = Not at all; 5 = Extremely). It is divided in two subscales: Positive Arousal Level and Negative Arousal Level.

Emotion Regulation Questionnaire (Gross and John, 2003): it is a self-administered scale measuring emotional control in daily life. It consists in 10 items on a 100-point visual analogue scale (0 = Strongly agree; 100 = Strongly disagree). It is divided in two subscales: cognitive reappraisal and expression suppression.

Somatic Strategies and Somatic Suppression scale (Gross et al., 2016): it is a self-administered scale measuring emotional regulation and suppression by physical means. It consists in 6 items on a 100-point visual analogue scale (0 = Strongly agree; 100 = Strongly disagree). It is divided in two subscales: emotional regulation via somatic strategies and suppression of somatic emotion manifestations.

Depression, Anxiety, and Stress Scale (Lovibond and Lovibond, 1995): it is a self-report measure of depression, anxiety, and stress. It consists in 21 items on a 4-point Likert scale (0 = Strongly disagree; 3 = Totally agree). It measures three dimensions: positive affectivity (Depression), physiological hyperarousal (Anxiety), and negative affectivity (Stress).

Beck Depression Inventory (Beck et al. 1961): it is a self-report measure of depression severity. It consists in 21 items on a 4-point Likert scale (0 = not at all; 4 = extremely).

# Supplementary Table 1: PRISMA Check-list

| **Section/topic** | **#** | **Checklist item** | **Reported on page #** |
| --- | --- | --- | --- |
| **TITLE** | | |  |
| Title | 1 | Identify the report as a systematic review, meta-analysis, or both. | 1 |
| **ABSTRACT** | | |  |
| Structured summary | 2 | Provide a structured summary including, as applicable: background; objectives; data sources; study eligibility criteria, participants, and interventions; study appraisal and synthesis methods; results; limitations; conclusions and implications of key findings; systematic review registration number. | 1-2 |
| **INTRODUCTION** | | |  |
| Rationale | 3 | Describe the rationale for the review in the context of what is already known. | 2-3 |
| Objectives | 4 | Provide an explicit statement of questions being addressed with reference to participants, interventions, comparisons, outcomes, and study design (PICOS). | 3-4, Table 1 |
| **METHODS** | | |  |
| Protocol and registration | 5 | Indicate if a review protocol exists, if and where it can be accessed (e.g., Web address), and, if available, provide registration information including registration number. | 4 |
| Eligibility criteria | 6 | Specify study characteristics (e.g., PICOS, length of follow-up) and report characteristics (e.g., years considered, language, publication status) used as criteria for eligibility, giving rationale. | 4, Table 1 |
| Information sources | 7 | Describe all information sources (e.g., databases with dates of coverage, contact with study authors to identify additional studies) in the search and date last searched. | 3-4 |
| Search | 8 | Present full electronic search strategy for at least one database, including any limits used, such that it could be repeated. | Table 2, Appendix 1 |
| Study selection | 9 | State the process for selecting studies (i.e., screening, eligibility, included in systematic review, and, if applicable, included in the meta-analysis). | 4-5, Fig.1 |
| Data collection process | 10 | Describe method of data extraction from reports (e.g., piloted forms, independently, in duplicate) and any processes for obtaining and confirming data from investigators. | 4-5 |
| Data items | 11 | List and define all variables for which data were sought (e.g., PICOS, funding sources) and any assumptions and simplifications made. | Table 3, Appendix 2 |
| Risk of bias in individual studies | 12 | Describe methods used for assessing risk of bias of individual studies (including specification of whether this was done at the study or outcome level), and how this information is to be used in any data synthesis. | Table S2, Table S3 |
| Summary measures | 13 | State the principal summary measures (e.g., risk ratio, difference in means). | Table 4 |
| Synthesis of results | 14 | Describe the methods of handling data and combining results of studies, if done, including measures of consistency (e.g., I^2^) for each meta-analysis. | N/A |

| **Section/topic** | **#** | **Checklist item** | **Reported on page #** |
| --- | --- | --- | --- |
| Risk of bias across studies | 15 | Specify any assessment of risk of bias that may affect the cumulative evidence (e.g., publication bias, selective reporting within studies). | N/A |
| Additional analyses | 16 | Describe methods of additional analyses (e.g., sensitivity or subgroup analyses, meta-regression), if done, indicating which were pre-specified. | N/A |
| **RESULTS** | | |  |
| Study selection | 17 | Give numbers of studies screened, assessed for eligibility, and included in the review, with reasons for exclusions at each stage, ideally with a flow diagram. | Figure 1 |
| Study characteristics | 18 | For each study, present characteristics for which data were extracted (e.g., study size, PICOS, follow-up period) and provide the citations. | Table 3 |
| Risk of bias within studies | 19 | Present data on risk of bias of each study and, if available, any outcome level assessment (see item 12). | 7-8, Table S2, Table S3 |
| Results of individual studies | 20 | For all outcomes considered (benefits or harms), present, for each study: (a) simple summary data for each intervention group (b) effect estimates and confidence intervals, ideally with a forest plot. | 5-7, Table 4 |
| Synthesis of results | 21 | Present results of each meta-analysis done, including confidence intervals and measures of consistency. | N/A |
| Risk of bias across studies | 22 | Present results of any assessment of risk of bias across studies (see Item 15). | 7-8, Table S2, Table S3 |
| Additional analysis | 23 | Give results of additional analyses, if done (e.g., sensitivity or subgroup analyses, meta-regression [see Item 16]). | N/A |
| **DISCUSSION** | | |  |
| Summary of evidence | 24 | Summarize the main findings including the strength of evidence for each main outcome; consider their relevance to key groups (e.g., healthcare providers, users, and policy makers). | 8-11 |
| Limitations | 25 | Discuss limitations at study and outcome level (e.g., risk of bias), and at review-level (e.g., incomplete retrieval of identified research, reporting bias). | 11-12 |
| Conclusions | 26 | Provide a general interpretation of the results in the context of other evidence, and implications for future research. | 12-13 |
| **FUNDING** | | |  |
| Funding | 27 | Describe sources of funding for the systematic review and other support (e.g., supply of data); role of funders for the systematic review. | 13 |

**Supplementary Table 2.** Risk of bias in within subject studies

| **Single-Case Reporting Guideline In BEhavioural Interventions (SCRIBE) check-list** | **Critchley et al., 2015** | **Edmonds et al., 2009** | **Fumoto et al., 2004** | **Gross et al., 2016** | **Lin et al., 2014** | **Park and Park, 2012** | **Stark et al., 2000** | **Tsuji, 2010** | **Van Diest et al., 2014** | **Yu et al., 2011** |
| --- | --- | --- | --- | --- | --- | --- | --- | --- | --- | --- |
| 1 Title Identify the research as a single-case experimental design in the title | NO | YES | NO | NO | NO | NO | NO | NO | NO | NO |
| 2 Abstract Summarize the research question, population, design, methods, including intervention/s (independent variable/s) and target, behavior/s and any other outcome/s (dependent variable/s), results, and conclusions | YES | NO | YES | YES | YES | YES | NO | YES | YES | YES |
| 3 Scientific background Describe the scientific background to identify issue/s under analysis, current scientific knowledge, and gaps in that knowledge base | YES | YES | YES | YES | YES | YES | YES | YES | YES | YES |
| 4 Aims State the purpose/aims of the study, research question/s, and, if applicable, hypotheses | YES | YES | YES | YES | YES | YES | YES | YES | YES | YES |
| 5 Design Identify the design (e.g., withdrawal/reversal, multiple-baseline, alternating-treatments, changing-criterion, some combination thereof, or adaptive design) and describe the phases and phase sequence (whether determined a priori or data-driven) and, if applicable, criteria for phase change | NO | YES | NO | YES | YES | NO | YES | NO | YES | NO |
| 6 Procedural changes Describe any procedural changes that occurred during the course of the investigation after the start of the study | NO | NO | NO | NO | NO | NO | NO | NO | NO | NO |
| 7 Replication Describe any planned replication | NO | YES | YES | YES | YES | NO | YES | NO | YES | NO |
| 8 Randomization State whether randomization was used, and if so, describe the randomization method and the elements of the study that were randomized | YES | YES | NO | NO | YES | NO | YES | NO | NO | NO |
| 9 Blinding State whether blinding/masking was used, and if so, describe who was blinded/masked | NO | NO | NO | NO | NO | NO | NO | NO | NO | NO |
| 10 Selection criteria State the inclusion and exclusion criteria, if applicable, and the method of recruitment | NO | YES | YES | NO | YES | YES | YES | NO | YES | YES |
| 11 Participant characteristics For each participant, describe the demographic characteristics and clinical (or other) features relevant to the research question, such that anonymity is ensured | NO | NO | NO | NO | YES | NO | NO | NO | NO | NO |
| 12 Setting Describe characteristics of the setting and location where the study was conducted | YES | NO | YES | NO | YES | YES | YES | YES | NO | YES |
| 13 Ethics State whether ethics approval was obtained and indicate if and how informed consent and/or assent were obtained | YES | NO | YES | YES | YES | YES | NO | NO | YES | YES |
| 14 Measures Operationally define all target behaviors and outcome measures, describe reliability and validity, state how they were selected, and how and when they were measured | YES | YES | YES | YES | YES | YES | YES | YES | YES | YES |
| 15 Equipment Clearly describe any equipment and/or materials (e.g., technological aids, biofeedback, computer programs, intervention manuals or other material resources) used to measure target behavior/s and other outcome/s or deliver the interventions | YES | YES | YES | YES | YES | YES | YES | YES | YES | YES |
| 16 Intervention Describe the intervention and control condition in each phase, including how and when they were actually administered, with as much detail as possible to facilitate attempts at replication | YES | YES | YES | YES | YES | YES | YES | YES | YES | YES |
| 17 Procedural fidelity Describe how procedural fidelity was evaluated in each phase | YES | YES | YES | YES | YES | YES | YES | NO | YES | NO |
| 18 Analyses Describe and justify all methods used to analyze data | YES | YES | YES | YES | YES | YES | YES | YES | YES | YES |
| 19 Sequence completed For each participant, report the sequence actually completed, including the number of trials for each session for each case. For participant/s who did not complete, state when they stopped and the reasons | YES | YES | NO | YES | YES | NO | YES | NO | YES | NO |
| 20 Outcomes and estimation For each participant, report results, including raw data, for each target behavior and other outcome/s | NO | YES | NO | NO | NO | NO | NO | NO | NO | NO |
| 21 Adverse events State whether or not any adverse events occurred for any participant and the phase in which they occurred | YES | NO | NO | YES | YES | NO | YES | YES | YES | YES |
| 22 Interpretation Summarize findings and interpret the results in the context of current evidence | YES | YES | YES | YES | YES | YES | YES | YES | YES | YES |
| 23 Limitations Discuss limitations, addressing sources of potential bias and imprecision | YES | YES | NO | YES | YES | YES | NO | NO | NO | YES |
| 24 Applicability Discuss applicability and implications of the study findings | YES | YES | NO | YES | YES | YES | NO | YES | YES | NO |
| 25 Protocol If available, state where a study protocol can be accessed | NO | NO | NO | NO | NO | NO | NO | NO | NO | NO |
| 26 Funding Identify source/s of funding and other support; describe the role of funders | YES | NO | NO | YES | NO | NO | NO | NO | YES | NO |

**Supplementary Table 3.** Risk of bias in pre-post studies

| **Quality Assessment Tool for pre-post designs** | **Gruzelier et al., 2014** | **Kharya et al., 2014** | **Lehrer et al., 2003** | **Sakakibara et al., 2013** | **Siepmann et al., 2008** |
| --- | --- | --- | --- | --- | --- |
| a. Was probability sampling used? (1) | 0 | 0 | 0 | 0 | 0 |
| b. Was sample size justified to obtain adequate power? (1) | 0 | 0 | 0 | 0 | 0 |
| **Subtotal (n/2)** | **0** | **0** | **0** | **0** | **0** |
| a. One pre-test or baseline and several post-test measures (2) or |  | 2 | 2 | 2 | 2 |
| b. Simple before-and-after study (1) | 1 |  |  |  |  |
| **Subtotal (n/2)** | **0.5** | **1** | **1** | **1** | **1** |
| Does the study employ a comparison strategy? An attempt to create or assess equivalence of groups at baseline by: |  |  |  |  |  |
| a. Matching group participants (2) or |  |  | 2 |  |  |
| b. Statistical control (1) or | 1 | 1 |  | 1 | 1 |
| c. None (0) |  |  |  |  |  |
| **Subtotal (n/2)** | **0.5** | **0.5** | **1** | **0.5** | **0.5** |
| a. Was the DV directly measured by an assessor? (1) | 1 | 1 | 1 | 1 | 1 |
| b. Were dependent variables either |  |  |  |  |  |
| i) Directly measured (2) or | 2 | 2 | 2 | 2 | 2 |
| ii) Self-reported (1) |  |  |  |  |  |
| c. Were dependent variables measured reliably (with reliability indices previously or for this study)? (1) | 1 | 1 | 1 | 1 | 1 |
| d. Were dependent variables measured validly (with validity assessments previously or for this study)? (1) | 1 | 1 | 1 | 1 | 1 |
| **Subtotal (n/5)** | **1** | **1** | **1** | **1** | **1** |
| a. Was (were) the statistical test(s) used appropriate for the main outcome and at least 80% of the others? (1) | 1 | 1 | 1 | 1 | 1 |
| b. Were p values and confidence intervals reported properly? (1) | 1 | 1 | 1 | 1 | 0 |
| c. If multiple outcomes were studied, were correlations analyzed? (1) | 0 | 0 | 1 | 0 | 0 |
| d. Were missing data managed appropriately? (1) | 1 | 0 | 1 | 1 | 0 |
| **Subtotal (n/4)** | **0.75** | **0.5** | **1** | **0.75** | **0.25** |
| a. Is attrition rate <30% (if no attrition code 1) (1) | 1 | 1 | 1 | 1 | 1 |
| **Subtotal (n/1)** | **1** | **1** | **1** | **1** | **1** |
| **Total (n/16)** | **0.6875** | **0.6875** | **0.875** | **0.75** | **0.625** |
| **Overall Validity Rating** | **Medium** | **Medium** | **High** | **Medium** | **Medium** |
